# Supplementary material for: Dietary Choline Alleviates High-Fat Diet-Induced Hepatic Lipid Dysregulation via UPRmt Modulated by SIRT3-Mediated mtHSP70 Deacetylation
Source: Int J Mol Sci. 2022 Apr 11;23(8):4204. doi: 10.3390/ijms23084204 (PMC9025889; doi:10.3390/ijms23084204)
Supplement: Supplementary file 1 [file ijms-23-04204-s001.zip › ijms-1607618-supplementary.pdf]

Table S1. Ingredients and proximate analysis of experimental diets.

Ingredients (g kg^{-1})	Control	HFD	HFD+Choline
Casein	360	360	360
Gelatin	80	80	80
Fish oil	30	45	45
Corn oil	30	45	45
Wheat flour	250	250	250
Vitamin C polyphosphate	10	10	10
NaCl	10	10	10
$Ca(H_2PO_4)_2 \cdot H_2O$	10	10	10
Vitamin premix	5	5	5
Mineral premix	5	5	5
Cellulose	209.5	179.5	178.5
Choline chloride	0.5	0.5	1.5
Proximate analysis (% dry matter basis)			
Moisture	7.15	6.98	7.05
Crude protein	40.1	39.8	40.9
Crude lipid	10.3	14.5	14.3
Crude ash	5.28	5.31	5.44
Choline chloride (mg kg^{-1})	563.4	578.9	1652.3

Vitamin premix (mg or IU per kg diet): retinylacetate 10,000 IU; cholecalciferol 1000 IU; all-rac-a-tocopheryl acetate 30 IU; menadione nicotinamide bisulfite 7; thiamine hydrochloride 6; riboflavin 3; pyridoxine hydrochloride 12; D-calcium pantothenate 30; niacin 50; biotin 1; folic acid 6; cyanocobalamine 0.03. Mineral mixture (mg per kg diet): $Ca(H_2PO_3)_2 \cdot H_2O$, 1000; $FeSO_4 \cdot 7H_2O$ 40; $ZnSO_4 \cdot H_2O$ 100; $MnSO_4 \cdot H_2O$ 40; $CuSO_4 \cdot 5H_2O$ 2; $CaIO_3 \cdot 6H_2O$ 3; Na_2SeO_3 0.05; $CoSO_4$ 0.05.

Table S2. The growth and diet coefficient result of the experimentation.

	Control	HFD	HFD+Choline
IBW, g/fish	3.78±0.05	3.78±0.03	3.85±0.01
FBW, g/fish	31.38±0.91a	35.10±0.14b	32.00±0.50ab
WG, %	728.60±0.15a	827.08±.061b	730.60±0.16a
SGR, %/d	3.02±0.03	3.18±0.01	3.02±0.03
FCR	1.15±0.03	1.08±0.03	1.14±0.01
Survival	98.32±2.96	98.66±2.30	97.33±3.21

Notes: Values are mean ± SEM (three replicate tanks, n = 3). The asterisk denotes means for Met deficiency or Met excess that are different from means for the adequate Met control ($P < 0.05$). IBW (g $fish^{-1}$), initial mean body weight; FBW (g $fish^{-1}$), final mean body weight; WG (weight gain, %) =100 × (final mean body weight − initial mean body weight) / initial mean body weight; SGR (specific growth rate, % d^{-1}) = 100 × (ln (FBW) − ln (IBW)) / days; FCR (feed conversion rate) = FI / (FBW − IBW + dead fish weight); Survival = 100 × (final fish number) / (initial fish number).

Table S3. Primers used for qPCR analysis.

Gene	Forward primer (5'-3')	Reverse primer (5'-3')	Accession no.
acca	GGGGTTTTCACGCTGCTTC	GGTTCTGATTGGGTCGTCCTG	JX992746
fas	AACTAAAGGCTGCTGGTTGCTA	GTACCGTCGTTCTTCAGCAA	JN579124
hsl	AAGAACCCGTTTGTATCTCCTCT	TGCTACGCAAATATC TGACGC	KJ588764
atgl	AGAGAGACCTGCCTGAACAC	CAGGAGGGAACAGACCACAA	KF614123
mgl	AGTGAGAGAGGACAGGGGAT	TTTGCCACTTGTGGGTATGC	KX980491
cpt1	ATGTGAGTGACTGGTGGGAG	CGCCGATATTGCAGGATAGC	JN579122
cpt2	CGAGATTGCCAGTTCCGAAG	GATTCACGGGCAGAGAGGTA	MG599811
acox1	TGGAGTGTGAATGCAGGGAT	ACGCACTTGTCTGTAGCTCT	MG599802
echs1	TGTTCGTCAATGCAGCTCAG	CTCTTTGATGTCAGCACCCG	MG599820
acads	AAGTAGGCTGCTTTGCTTTGAGTGAG	AATTGGTGATCCAGGCTTTGGTG	MG599806
acadm	GCAGAAGGAGTTCCAGGAGGTGTC	CAGCAATAATGACCGGCATTTGTC	MG599804
acadl	TAACGGCTGGATGAGTGACCTGG	GCTATTATCAACCGCTCCTGTGGC	MG599805
hadha	GCTTGACGACAGGTTTCGGGTTT	TGTTTAGCTGCTTATCCTTTGAT-TTGG	MG599815
hadhb	CGACATCGACGTGTTCGAGTTCC	GCCATACTGCCCTCCCTCCTTCT	MG599812
sirt3	GTTTGCCAGCTTATCCGGAG	TCCGCGTAGTCATTCCTCTC	XM_027153054
mthsp70	ATGCAACCAAACGCCTGATT	TGGTACAGTCACGACAGCAT	XM_027135492
hsp10	TTCCTGCCAATGTTTGACCG	GCGTTACAGCTCCAGTCTTG	XM_027166043
lonp1	CCCGCTGGTTCTCATTGATG	TTTCCTGAGCCACATAGCCA	XM_027159514
clpp	GCCATCTAGCGGTCAGTTTG	CACCTCGACCTGTCTGTTCT	XM_027171871
tomm40	CGTTCTTCTCGGATCTGGGA	TGCTTGCTTCAAACTCCACC	XM_027154188
tomm70	AACAGCCCGTCTCAAGTACA	TGTGGCATTATCAGGCTCCA	XM_027146788
timm22	GTGACCCGGAGAAGTGTGTA	TTCTGCCGTCCTCTGATTGT	XM_027165350
timm23	CATAACTGGGACCGCTTTCG	TGTTAACGTCCCAGCAGCTA	XM_027140359
β-actin	GGACTCTGGTGATGGTGTGA	CTGTAGCCTCTCTCGGTCAG	EU161066
rpl7	GGCAAATGTACAGGAGCGAG	GCCTTGTTGAGCTTGACGAA	KP938522
hprt	ATGCTTCTGACCTGGAACGT	TTGCGGTTCAGTGCTTTGAT	KP938523
tuba	TCAAAGCTGGAGTTCTCGGT	AATGGCCTCGTTATCCACCA	KP938526
b2m	GCTGATCTGCCATGTGAGTG	TGTCTGACACTGCAGCTGTA	KP938520
ubce	TCAAGAAGAGCCAGTGGAGG	TAGGGGTAGTCGATGGGGAA	KP938524
tbp	AGCAAAGAGTGAGGAGCAGT	ACTGCTGATGGGTGAGAACA	KP938525
gapdh	TTTCAGCGAGAGAGACCCAG	ATGACTCTCTTGGCACCTCC	KP938521
18s rrna	AGCTCGTAGTTGGATCTCGG	CGGGTATTCAGGCGAGTTTG	KP938527
elfa	GTCTGGAGATGCTGCCATTG	AGCCTTCTTCTCAACGCTCT	KU886307

A

siRNA	Position	SS Sequence	AS Sequence
si-Sirt3-1	142	CCGAGACGGUGUACUCUAAAU	UUAGAGUACACCGUCUCGGUA
si-Sirt3-2	222	CAGGAAGGUUUCAGACAAAUU	UUUGUCUGAAACCUUCCUGUG
si-Sirt3-3	713	CGAAGUUAGUGGAAGCACAUG	UGUGCUUCCACUAACUUCGUA

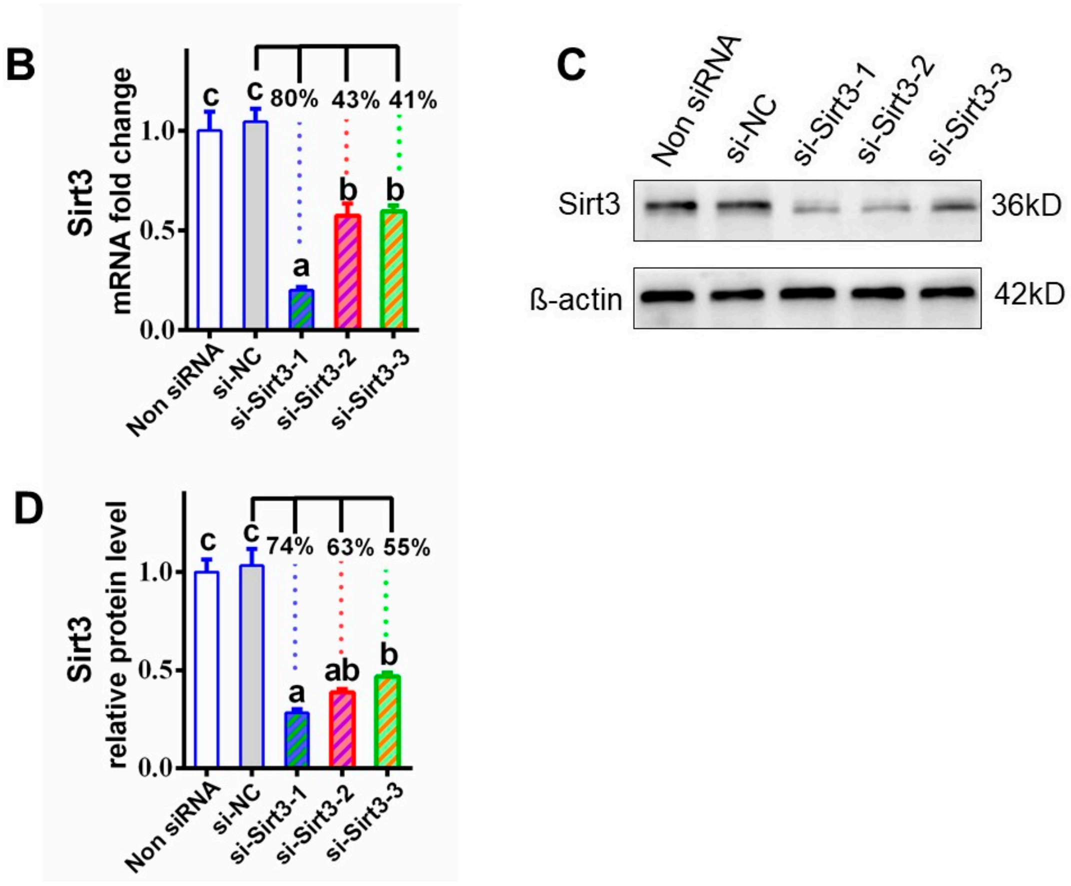

Figure S1. Primers for si-Sirt3 and confirmation for inhibitory effect of si-Sirt3 on expression of Sirt3 at both protein and gene levels.

A

Mutation site	Forward primer (5'-3')	Reverse primer (5'-3')
**K493R**	ATCCAGGCAGGGGCAACCCAGA GGATGAATGA	GTTGCCCCTGCCACTCAGGGACTG CAGACC
**K507R**	AGCCAATTAAACAGGACAAATGTG AAATTCCTCGAAC	GTCCTGTTTAACAAGGCCACCAACT TATG

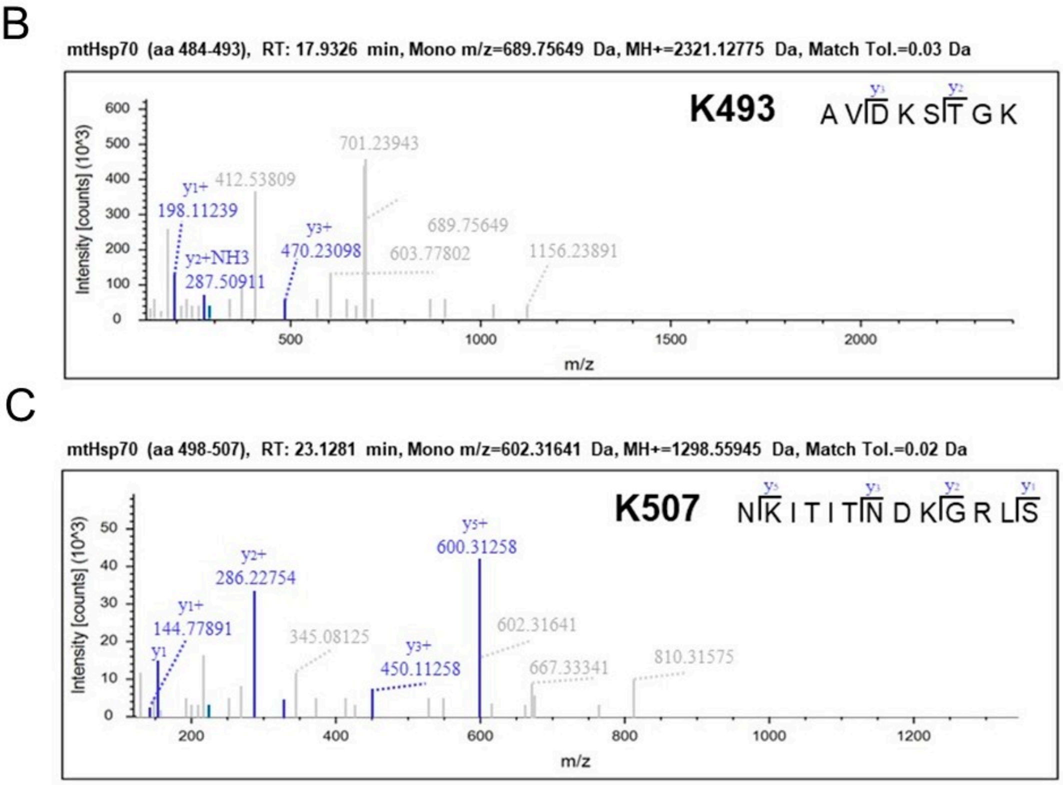

D

yellow catfish 490-511	AVDKSTGKENKITITNDKGRLS
zebrafish 492-513	AADKSTGKQNKITITNDKGRLS
mouse 490-511	ATDKSTGKANKITITNDKGRLS
cow 490-511	ATDKSTGKANKITITNDKGRLS
chiken 493-514	AVDKSTGKENKITITNDKGRLS
frog 491-512	AVDKSSGKKNKITITNDKGRLS

Figure S2. Confirmation for acetylation sites of mtHsp70 Acetylation sites in *P. fulvidraco*. (A) Primers used for mutations of lysine residue. (B) Identification of mtHsp70 acetylation sites by liquid chromatography-mass spectrometry of hepatocytes of *P. fulvidraco*. (C) Alignment of the protein sequences of mtHsp70 homologues among various species. The red K indicates the putative acetylated lysine resides of mtHsp70.
